# Reinforcement and Compensatory Mechanisms in Attention-Deficit Hyperactivity Disorder: A Systematic Review of Case-Control Studies

**DOI:** 10.7759/cureus.13718

**Published:** 2021-03-05

**Authors:** Mrinaal Valmiki, Peter Fawzy, Surbhi Valmiki, Mohamed A Aid, Ali R Chaitou, Maria Zahid, Safeera Khan

**Affiliations:** 1 Psychiatry, California Institute of Behavioral Neurosciences & Psychology, Fairfield, USA; 2 Neurological Surgery, California Institute of Behavioral Neurosciences & Psychology, Fairfield, USA; 3 Obstetrics and Gynecology, California Institute of Behavioral Neurosciences & Psychology, Fairfield, USA; 4 Intensive Care Unit, California Institute of Behavioral Neurosciences & Psychology, Fairfield, USA; 5 Internal Medicine, California Institute of Behavioral Neurosciences & Psychology, Fairfield, USA

**Keywords:** attention-deficit hyperactivity disorder, reinforcement, reward processing

## Abstract

Attention-deficit hyperactivity disorder (ADHD) is a neuropsychological disorder that causes inattentiveness, hyperactivity, and impulsiveness in patients. Ventral striatal hypo-responsiveness, orbitofrontal cortex, and dopaminergic status in the brain are related to the pathogenesis of ADHD. Reinforcement tasks by monetary incentive delay (MID) was shown to produce more responsiveness in patients. In this study, we reviewed how reinforcement interventions and compensatory mechanisms affect the behavior of ADHD patients. This systematic review was undertaken as per the Preferred Reporting Items for Systematic Review and Meta-Analysis guidelines, and PubMed database was used for literature search.

The quality appraisal was completed using the Newcastle-Ottawa scale, and nine case-control studies were included in this systematic review. A total of 976 participants were included, with 493 cases and 330 controls. The studies included discuss reinforcement, attention networks, and compensatory mechanisms. Our review concludes that reinforcement improves responsiveness to gain and loss of rewards in ADHD patients. Reward processing is selectively associated with the salience network. While ADHD, predominantly the inattentive type, is insensitive to stimuli, ADHD combined type and controls showed similar responsiveness. The right visual cortex may also be related to compensatory mechanisms in ADHD. As we only included case-control studies from the last eight years, in the English language, we might have missed some relevant studies related to this research. Because the included studies have a relatively small sample size, we recommend future studies to explore larger cohorts of patients to improve the reliability of findings pertinent to this field.

## Introduction and background

Attention-deficit hyperactivity disorder (ADHD) is a neurological and behavioral disorder that affects not only the person but the entire family, including parents and extended family of parental siblings and grandparents. It tests the limits of the family’s ability to be supportive, understanding, and loving [1].

ADHD is a heterogeneous group of neurodevelopmental disorders, defined and diagnosed with the help of the Diagnostic and Statistical Manual of Mental Disorders (DSM-MD). It is the most prevalent psychiatric disorder in adolescents, affecting 5.29% of adolescent children worldwide and 9.4% of children in the United States [2-5]. It is characterized by the inadequacy of various neuropsychiatric processes and the related tracts of nerves, which lead to paucity in the venture, attention, motor operations, inhibition, motivation, and reward processing. Patients with ADHD have a preference for minor prompt rewards over major, belated rewards, make more unsound decisions to attain rewards, and are exceptionally responsive to positive reinforcement [6-8].

The hypothesis underlying the pathogenesis of ADHD revolves around the reward processing areas of the brain, which are the ventral striatum and orbitofrontal cortex (OFC), as well as the hypo-dopaminergic status of an individual. The dysfunctional interaction between these results in disarranged sensitivity toward reinforcement, such as rewards, which can be either decreased or increased in reward centers [7,9-11]. The goal-oriented restrictions of behavior can also be disarranged due to disarrangement in reward-related behavior. As the OFC is the center of goal-oriented behavior [12-14], lesions of the OFC are correlated to impulsive choices and maladjusted behavior in ADHD patients. This can account for the probability that OFC dysfunction might result in an ineffective understanding of the reward’s value and, thus, impaired goal-oriented behavior [15,16].

Discounting is a part of an individual’s nature and causes them to devalue rewards that are either delayed, uncertain, or require more effort. This phenomenon is assumed to be strongly linked to dopamine function [17]. Several tasks-based functional magnetic resonance imaging (fMRI) studies devoted to mapping reward circuits have identified the key structures related to reward processing, which include dopaminergic midbrain, ventral striatum, anterior cingulate cortex, and OFC [18]. These studies mostly anoint a monetary incentive delay (MID) task and most regularly report attenuation response of ventral striatum during reward processing [19]. Some studies have suggested that the striatal threshold may be distributed by the profound activity of the amygdala, which causes an aberrant response to stimuli, which is thought to be aggravated in ADHD. This could disrupt learning because the ventral striatal threshold is regulated by inputs from the amygdala [20-22].

ADHD has three subtypes: ADHD-I, predominantly inattentive type (45% of all ADHD patients); ADHD-HI, predominantly hyperactive-impulsive type (21% of all ADHD patients); and ADHD-CT, combined type (34% of all ADHD patients) [3,23]. Reinforcement is markedly related to motivation, and the patients usually lack effort and motivation, resulting in low levels of performance. Therefore, they cannot depend solely on their elemental motivations without external implements [24,25]. Emotional-motivational dysfunction could be the cause of symptoms of ADHD. However, the three subtypes display differences regarding this dysfunction. ADHD-CT and ADHD-HI have been shown to be responsive to this, while ADHD-I appears to be less responsive. This could be the cause of hypersensitivity impulsiveness, but not inattentiveness [26]. Compensatory mechanism can be associated with visual processing in the right occipital region that substitutes for lack of attention in patients [27]. A biofeedback mode (i.e., neurofeedback) and cognitive training are being implemented as non-pharmacological treatment. Slow cortical potential neurofeedback is a prime route toward maintaining symptoms of ADHD and better regulation of brain activity [28].

This review focuses on exploring the mechanisms by which ADHD patients respond to applied reinforcement in the form of monetary incentive tasks across the different subtypes of ADHD, compensatory mechanisms involved, and how attention and execution networks function in ADHD patients that make them perform in a different way than normally developing individuals.

## Review

Methods

Protocol

This systematic review was conducted and reported in the accordance with the Preferred Reporting Items for Systematic Review and Meta-Analysis (PRISMA) [29].

Inclusion/Exclusion Criteria

The literature search was done to identify studies that defined ADHD, reward processing, and reinforcement in adolescent children. The criteria used to search for eligible studies included the following: (1) reinforcement in male adolescents with ADHD, and (2) reward processing in male patients with ADHD. The studies that reported other illnesses and those that included female patients along with male patients or only female patients were excluded as they were outside the scope of the extant study. The case-control studies discussing reward processing and reinforcement in adolescent males with ADHD and subtypes of ADHD were considered for inclusion, while other types of clinical studies were excluded.

Search Strategy

A methodical search of the database, PubMed [30], was conducted on November 30, 2020. The search for relevant studies using generic keywords (“Attention deficit hyperactivity disorders” AND “reinforcement” OR “reward processing”) was done and 2,263 studies were identified. The relevant Medical Subject Headings (MeSH) terms and keywords “attention deficit hyperactivity disorders,” “reinforcement, psychology,” “physiopathology,” “psychology,” “pathology,” and “diagnosis” were used in various combinations using Boolean operators like “AND” and “OR,” and 2,882 relevant studies were identified with a total of 5,145 studies. The inclusion/exclusion criteria were applied and records from January 2012 to November 2020 were identified. The search was extended to studies published in the English language for human participants. The searched articles were managed in Microsoft Word. Gray literature was not included in this study in accordance with inclusion/exclusion criteria. Table 1 and Table 2 display the results of the search strategy with the respective keywords.

**Table 1:** Database search results with regular keywords.

Keywords	Total articles	2012-2020	Inclusion/Exclusion	Full-text
(“Attention deficit hyperactivity disorders” AND “reinforcement” OR “reward processing”)	2,263	1,870	282	172

**Table 2:** Displaying the entire MeSH search strategy. MeSH, Medical Subject Headings Note: The data shown in the table display results obtained for each keyword combination individually. Thus, these results still contain duplicates which were removed later.

MeSH terms	Total articles	2012-2020	Inclusion/Exclusion	Full-text
(“Reinforcement, Psychology”[Mesh]) AND (“Attention Deficit Disorder with Hyperactivity”[Mesh])	542	229	62	36
(“Attention Deficit Disorder with Hyperactivity/psychology”[Mesh]) AND (“Attention Deficit Disorder with Hyperactivity/pathology”[Mesh])	101	57	32	18
(“Attention Deficit Disorder with Hyperactivity/physiopathology”[Mesh]) AND (“Attention Deficit Disorder with Hyperactivity/diagnosis”[Mesh])	1106	522	216	100
(“Attention Deficit Disorder with Hyperactivity/pathology”[Mesh]) AND (“Attention Deficit Disorder with Hyperactivity/diagnosis”[Mesh])	137	86	41	26
(“Attention Deficit Disorder with Hyperactivity/physiopathology”[Mesh]) AND (“Attention Deficit Disorder with Hyperactivity/psychology”[Mesh])	996	493	212	90

Data Extraction

All titles, abstracts, and full-text articles were screened by two reviewers independently, MV and PF. The items extracted from each study included year of publication, sample size, age range, response rate, study design, and study outcome. The studies gathered by one reviewer were also scrutinized by other reviewers for accuracy and eligibility. In case of dissidence, conflicts were resolved by a mutual discussion on the study in question.

Bias Evaluation and Data Explication

The quality appraisal was done using the Newcastle-Ottawa scale for the included case-control studies. Only moderate-to-high quality studies were included in the final analysis. Table 3 shows the results of the quality appraisal of the included studies [31-39].

**Table 3:** Quality appraisal of studies included in this analysis. _Selection: (1) Case definition adequate?; (2) representativeness of the case; (3) selection of controls; (4) definition of controls (1 point for each question asked for selection)_ _Comparability: 1 point if only cases were studied; 2 points if both cases and controls were studied and compared_ _Exposure: (1) Ascertainment of exposure; (2) the same method of ascertainment for controls; (3) non-response rate (1 point for each statement asked regarding exposure)_

References	Tool used	Selection				Comparability	Outcome			Total
		1	2	3	4	2 (points)	1	2	3	9
Abramov et al. 2019 [30]	Newcastle-Ottawa scale	1	1		1	1	1	1		6
Chevrier et al. 2019 [38]	Newcastle-Ottawa scale	1		1	1	1	1	1		6
Tegelbeckers et al. 2018 [37]	Newcastle-Ottawa scale	1	1	1		2	1	1		7
Von Rhein et al. 2017 [32]	Newcastle-Ottawa scale	1	1	1	1	2	1	1		8
Chronaki et al. 2017 [36]	Newcastle-Ottawa scale	1		1	1	1	1	1		6
Oldehinkel et al. 2016 [31]	Newcastle-Ottawa scale	1	1	1	1	2	1	1		8
Kappel et al. 2015 [35]	Newcastle-Ottawa scale	1	1	1	1	2	1	1		8
Gong et al. 2014 [34]	Newcastle-Ottawa scale	1	1	1	1	2	1	1		8
Paloyelis et al. 2012 [33]	Newcastle-Ottawa scale	1	1	1	1	1	1	1		7

Results

Search Outcome

**Figure 1:**
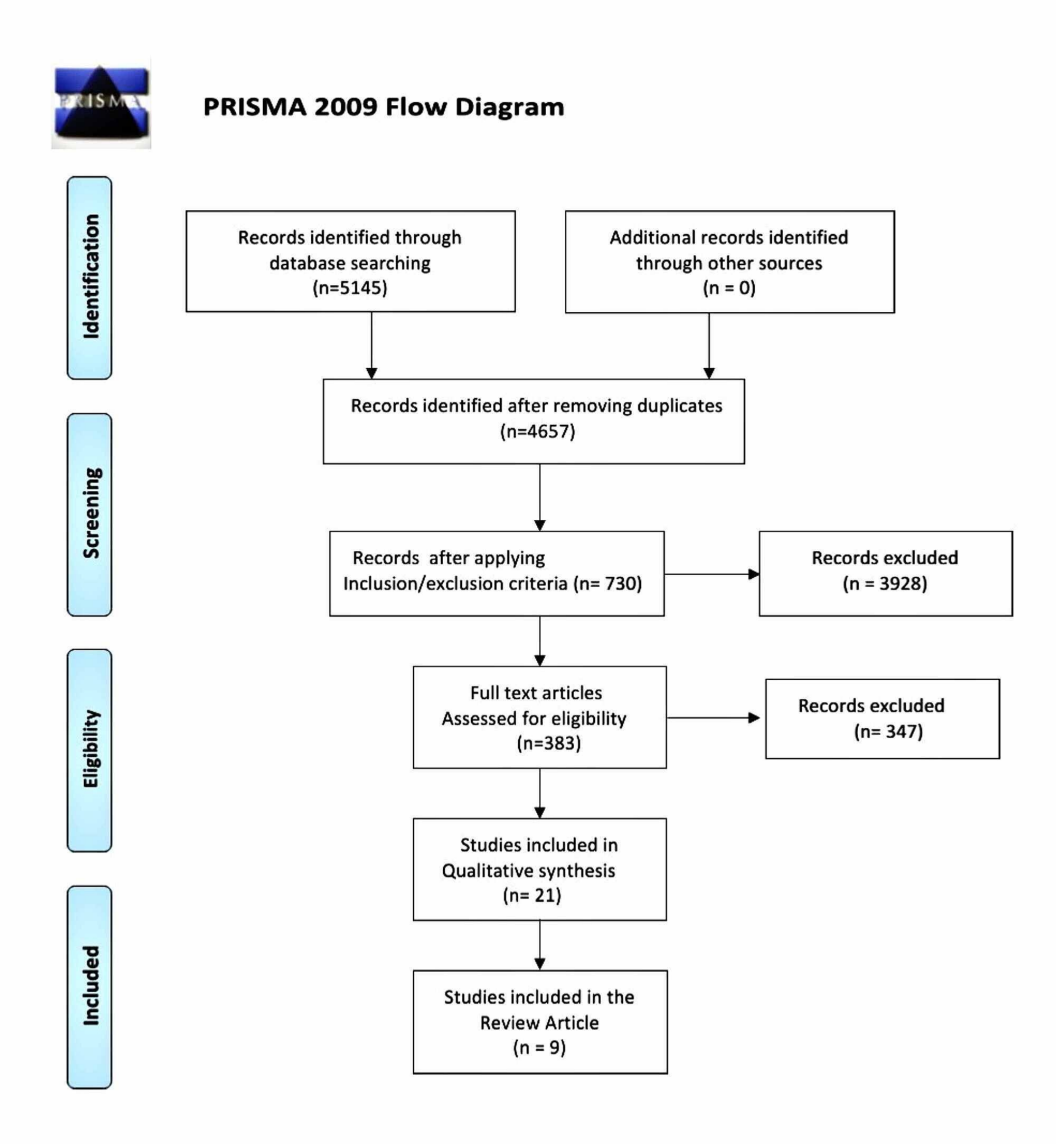
PRISMA flow diagram. PRISMA, Preferred Reporting Items for Systematic Review and Meta-Analysis

A total of 5,145 papers were identified through field search of the database and controlled vocabulary, that is, MeSH; 488 duplicates were removed using EndNote Basic and 4,657 articles were screened for eligibility regarding inclusion/exclusion criteria, and 730 studies were identified (383 full-text articles) which were screened through title and abstract for compatibility in the ongoing analysis. A total of 21 relevant studies were assessed for quality appraisal and nine moderate-to-high quality case-control studies were included in this systematic review. Figure 1 demonstrates the PRISMA flow diagram and the steps taken in conducting the search for the present review.

The nine articles included here were published in peer-reviewed journals from 2012 to 2020 and discussed reinforcement and reward processing in patients with ADHD and the subtypes of ADHD. One article also discussed possible compensatory mechanisms in ADHD patients. The sample size of the studies included was small ranging approximately 14-32 cases, and the sample size was the same for the control group. Only two studies had larger sample sizes of 150 cases and 48 controls in one and 169 cases and 122 controls in another. ADHD was explored in all the articles and patients were diagnosed using the Diagnostic and Statistical Manual of Mental Disorder (DSM-5).

A total of 976 participants were included, of which 493 participants were ADHD patients and 330 were controls. The rest of the participants were subthreshold cases and unaffected siblings of the cases from one study. The study findings of the included studies are summarized in Table 4 [32-40].

**Table 4:** Findings of the studies included in the analysis. ADHD, attention-deficit hyperactivity disorder; ANT, attention network testing; ADHD-CT, attention-deficit hyperactivity disorder, combined type; ADHD-I, attention-deficit hyperactivity disorder inattentive type; IVRT, RT, response time; fMRI, functional magnetic resonance imaging; OFC, orbitofrontal cortex; TD, typically developed; DMN: default mode network; MID, monetary incentive delay; FRN, feedback-related negativity; LPP, late positive potential; BOLD, brain oxygen level-dependent

References	Study type	Study characteristics	Method	Outcome
Abramov et al. (2019) [40]	Case-control	n = 19 (ADHD) n = 20 (control) age = 10-13 years	ANT in terms of modulated alertness under the effect of neutral stimuli and no stimuli, RT, IVRT, and response accuracy (AC) testing	ADHD cases displayed lesser effectivity of alertness and execution network respecting IVRT and AC. ANT, in all conditions, showed more asymmetry in the ADHD group than the control group
Chervrier et al. (2019) [39]	Case-control	n = 14 (ADHD) n = 14 (control) age = 12-17 years	Stop signal task, elementary choice reaction time, and subsidiary stop task. A stop signal followed by go stimuli instructed them to stop only when they see a stop signal and not to wait for it. fMRI was used for network screening	ADHD patients displayed profound activity of the amygdala and altered correlation among multiple neurotransmitter nuclei, altered control of dopamine, preventing thresholding on post-error slowing
Tegelbeckers et al. (2018) [38]	Case-control	n = 19 (ADHD) n = 20 (control) age = 12-16 years	Decision-making task under conditional stimuli, associated with large and small monetary rewards fMRI used for data collection	Among all participants, a significant signal increase to major versus minor awaited rewards in OFC. Responses were significantly enhanced in ADHD patients than TD controls
Von Rhein et al. (2017) [33]	Case-control	n = 150 (ADHD) n = 48 (control)	Independent component analysis applied to MID task; fMRI used for network observation	ADHD was associated with decreased working connection of salience and executive control network, as well as in the periphery of the brain
Chronaki et al. (2017) [37]	Case-control	n = 32 (ADHD) n = 31 (control) age = 10-16 years	Electrophysiological electronic-MID task performed under three conditions: (1) positive reinforcement, (2) negative reinforcement, and (3) neutral	No significant difference with neutral, but hypersensitivity to positive (marginally negative) reinforcement was observed in ADHD
Oldehinkel et al. (2016) [32]	Case-control	n = 169 (ADHD) n = 122 (control) n = 89 (siblings of cases) n = 64 (subthreshold) age: 17.7 years mean age	Resting fMRI was used to assess the resting-state functional connectivity of networks related to reward processing	No significant ADHD-related alteration in functional connectivity of the salience network was observed. Yet, alterations were observed in DMN and the frontoparietal networks
Kappel et al. (2015) [36]	Case-control	n = 30 (ADHD) n = 30 (controls)	MID task performed by participants. fMRI was used to compare ventral striatal structure and function in reward anticipation	Decreased ventral striatal activity was observed in reward prospect in unmedicated adults, but was absent in children who were never medicated
Gong et al. (2014) [35]	Case-control	n = 16 (ADHD-CT) n = 15 (ADHD-I) n = 15 (controls)	Participants performed a gambling task under feedback conditions: (1) large loss, (2) small loss, and (3) gain. FRN and LPP components of brain potential were recorded and analyzed	ADHD-CT and controls, larger losses evoke more negative FRN amplitude, suggesting brain sensitivity to punishment was absent in ADHD-I. LPP amplitude was also larger in ADHD-CT than ADHD-I. ADHD-CT showed intact brain sensitivity to punishment as controls
Paloyelis et al. (2012) [34]	Case-control	n = 29 (ADHD-CT) n = 30 (controls)	MILT was performed by participants. fMRI used for data collection focused on ventral striatum and caudate nucleus	ADHD-CT was the same as controls in terms of BOLD fMRI in reward anticipatory cue in the ventral striatum

Discussion

ADHD is a disorder of childhood and adolescence, but some symptoms persist through adulthood. Several mechanisms and areas of the brain are speculated to be involved in the development of signs and symptoms of ADHD. In this review, we aimed to bring them to light and improve the understanding of the various aspects of this disorder.

Reward Processing Networks

From previous studies, we know that reward processing is related to four networks: (1) the default mode network (DMN), (2) the frontoparietal network, (3) the lateral visual network, and (4) the salience network.

**Figure 2:**
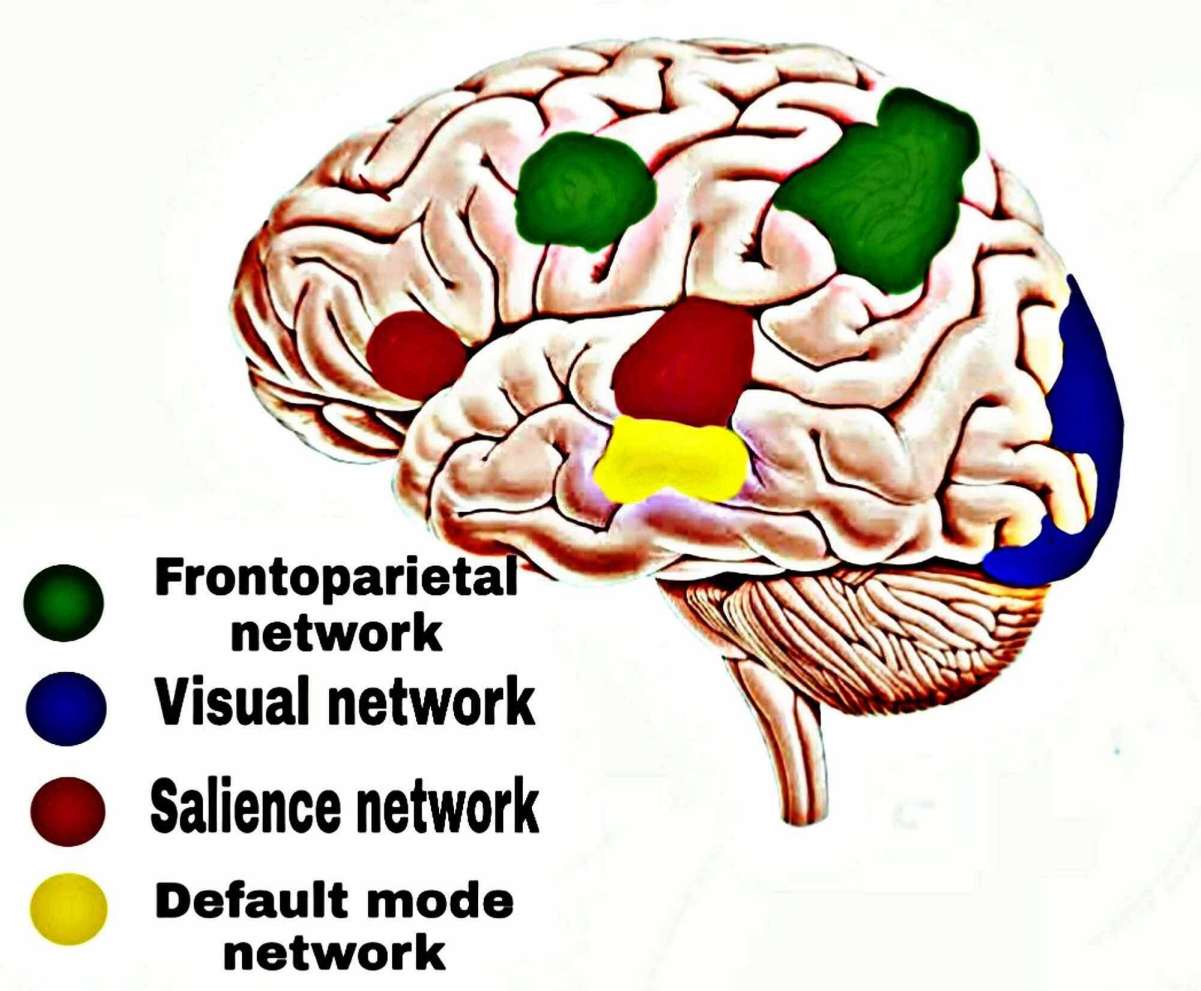
Location of the reward processing networks.

However, Oldehinkel et al. [31] showed no association of dynamic connectivity in reward exclusive networks, including nucleus accumbens (NAcc) and anterior cingulate cortex (ACC), which are parts of the salience network. Of note, the authors of this study observed that functional connectivity in parts of the DMN and frontoparietal network increased with higher inattention scores. These networks showed average loading in almost all task aspects, implying that they had a grossly supportive role in the execution of the task and were comprehensively unrelated to reward processing.

von Rhein et al. [32] also identified these four networks by assessing functional connection during resting state. They observed that out of these networks, three were related to general reward-independent task response and one was specifically related to reward processing. They suggested that ADHD might be concerned with the switched usage of fairly sound reward processing networks. They did not observe any ADHD-related effect in resting-state functional connectivity between the functional units of the salience network; however, they observed inattention restrained resting-state functional connectivity with the DMN and the frontoparietal network, which were related to general task processing. This was also in line with the above-mentioned study. They also observed that the salience network was selectively allied with rewarded task aspects, whereas other networks were only related to general cognitive processing. Compared to controls, cases showed altered functional connectivity in the salience network; however, other networks showed no difference in both cases and controls. Figure 2 shows the location of the reward processing network [31,32].

Attention-Deficit Hyperactivity Disorder Subtypes and Controls

ADHD patients are divided into three types: (1) ADHD-I, predominantly inattentive; (2) ADHD-HI, predominantly hyperactive-impulsive; and (3) ADHD-CT, combined type.

Paloyelis et al. [33] studied ADHD-CT and controls and reported no difference in terms of cue-elicit (gain or loss) neural activation in the ventral striatum. ADHD-CT patients exhibit relatively high brain oxygen level-dependent fMRI response, and both groups showed a similar increment in anticipatory activation and lesser response time with incentive significance without considering incentive demeanor. There was no difference in task performance in both ADHD-CT cases and controls. These findings challenge the concept of deficit neural activity in cases for incentive in the ventral striatum.

Gong et al. [34] showed similarity in response in ADHD-CT and typically developed controls, with both groups showing enhanced feedback-related negativity (FRN) to large losses than smaller losses and gains. They also reported that children with ADHD-CT and controls were not different in feedback FRN amplitudes. Therefore, similar to controls, ADHD-CT cases were also sensitive to punishment and the magnitude of punishment. This FRN response was absent in ADHD-I under all circumstances, including larger punishment. This explains their behavior of cognitive attention-deficit, that is, they are less alert. ADHD-CT patients are both impulsive and inattentive but they are also competitive, which may be associated with their enhanced sensitivity to negative outcomes. This can be considered a compensatory feature of these patients; therefore, they display sturdy FRN response despite inattention. Late positive potential (LPP) score was also higher in ADHD-CT than ADHD-I, hence, ADHD-CT shows competitiveness leading to greater sensitivity.

**Figure 3:**
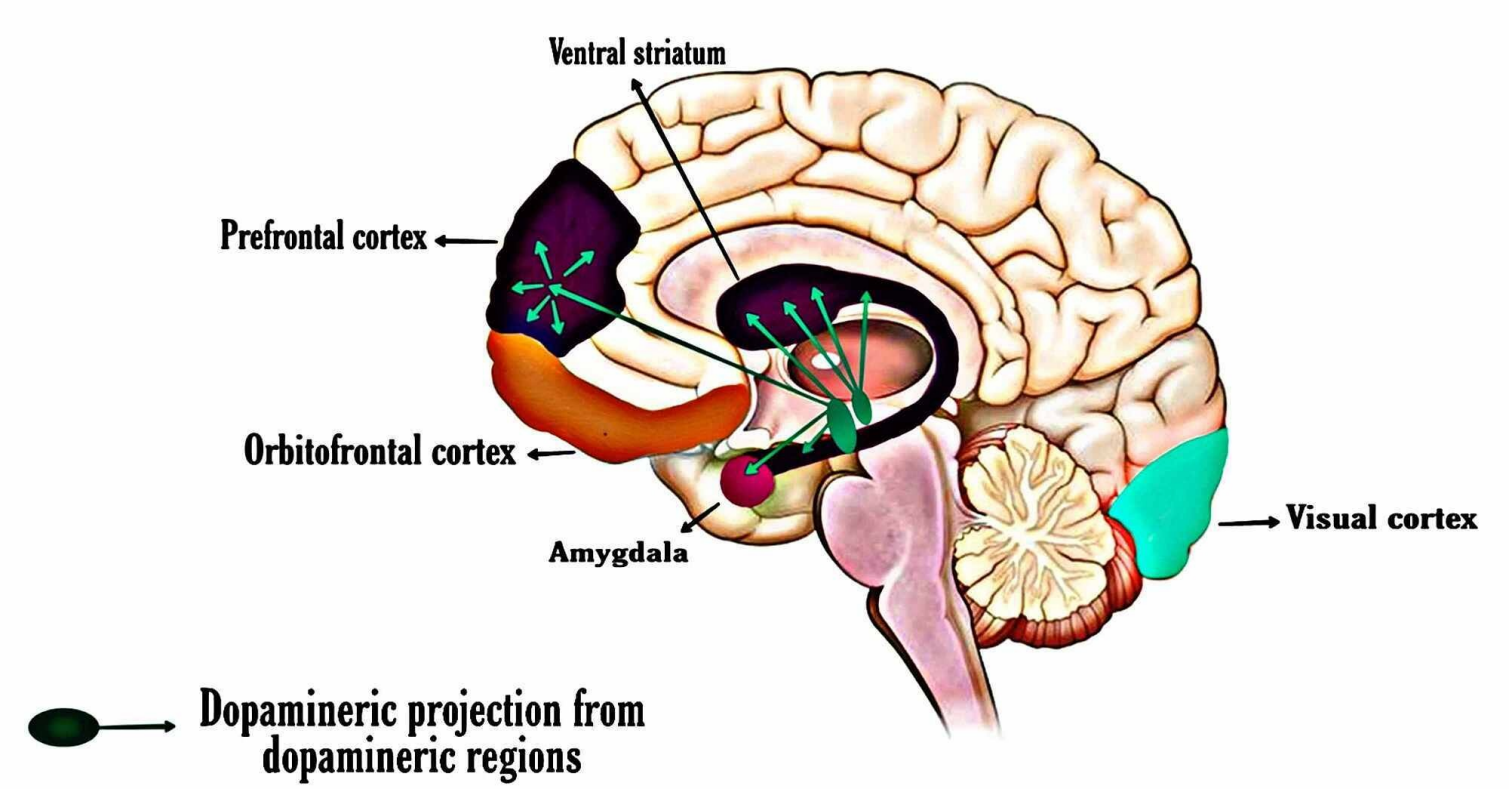
ADHD-related regions of the brain, the dopamine pathway. ADHD, attention-deficit hyperactivity disorder

Kappel et al. [35] demonstrated that during reward anticipation processing, decremental ventral striatal activity was seen in unmedicated adults with ADHD-CT but not in drug-naive children. (Although the patients were kept drug-free for two weeks, the long-term effects of the drugs are unclear.) Hence, ADHD-CT children and adults seem to act differently to expected rewards. ADHD-CT children did not demonstrate diminished activity of the ventral striatal during reward expectation and they acted similar to healthy controls; both of these groups reacted faster during gain and loss trials than neutral trials. This finding is in line with the above-mentioned studies. Reward-related learning impairment in children seems to be associated with prematurity of prefrontal structures included in executive management, and impairment in adults seems to be related to a lack in the unification of reward tidings. This paucity may reflect decreased dopaminergic inputs from the midbrain via the ventral striatum [40].) Reward-related ventral striatal hypo-responsiveness was also seen in a drug-naive homogeneous sample of patients with ADHD-I but not in ADHD-CT. Ventral striatal responsiveness might be free of qualitative ventral striatal disparity. Figure 3 shows the position of the prefrontal cortex and ventral striatum [33-35].

Reinforcement and Orbitofrontal Cortex

Using the e-MID task, a study by Chronaki et al. [36] tested the effect of reinforcement under three different circumstances: (1) gain (positive reinforcement), (2) loss (negative reinforcement), and (3) neutral.

The electrophysiological brain response showed that the amplitudes were convincingly high in monetary gain than neutral conditions for cases but not for controls during the awaiting and targeting stage of reinforced tasks. This was somewhat true for negative reinforcement as well. This confirmed the sensitivity to anticipatory incentives in ADHD cases, their attention to both, gain and loss, was enhanced and led to better management of motivational task-related stimuli. This study provided no evidence of ADHD-related hyposensitivity to tasks predicting gain or loss. Instead, cases showed a greater neural response in gain conditions. They found that there was greater neural activity in the ventral striatum and ACC in the adolescent with ADHD compared to controls, that is, cases appeared to be hypersensitive rather than hyposensitive to expected rewards.

This is in contradiction to another study by Tegelbackers et al. [37] that concluded that ADHD patients were slower than controls and that their response time was more than controls while performing tasks. Although the response was greater to a higher value of gain than lower value conditions in both the groups. From previous studies, it is known that the OFC plays an essential role in goal-directing behavior and decision-making. In their study, it was seen that OFC signaling to expected rewards was different in cases and controls, specifically more to larger rewards than smaller ones. fMRI analysis showed that there was enhanced signaling in OFC and diminished striatal response which may be the cause of imbalance between neural networks controlling reward-directing behavior, which may be the reason of reward-associated impulsiveness and hyperactivity in cases, suggesting that dopamine dysfunction is not the solitary contributor of reward-related behavior.

A study by Chevrier et al. [38] analyzed error detection and post-error deceleration that occurs during the response to a task (stop signal task) in cases and controls. Profound amygdala activity on error detection was equivalent to that observed during tasks that used emotional stimuli, and related analysis were persistent with activity halt initiation of post-error deceleration in ADHD patients. Whereas in healthy controls, deactivation in the ventral striatum was observed in error ascertain and post-error deceleration in the right frontoparietal region, which is known to be activated in the response phase; however, this finding was not significant in ADHD cases. However, the impedance of pre-frontal and not parietal reactance-phase activity with lack of parietal netting in task associate processes observed in AHDH patients. Intense activity altered intercorrelation among various neurotransmitter nuclei was persistent with the switched match of the curb of dopamine in the cases.

Reinforced learning impacts in the striatum and between neurotransmitter nuclei throughout an expected error are vital in relation to basic and overturned development as the unified function of the system sharply controls how all functional networks work rooted in previous experiences. ADHD cases and controls displayed a highly noticeable pattern of activity and intersubject association on error detection and more median post-error reaction time. Reinforced learning drives basic development, and diverted reinforced learning most likely drives the flourishing of ADHD patient’s brains. Figure 3 displays the OFC, the ventral striatum, the amygdala, and the dopaminergic outputs [36-38].

Compensatory Mechanisms

Abramov et al. [39] studied the interhemispheric attention network and found some asymmetry in ADHD cases than controls associated with the efficiency of the alerting network. According to their observations, the ADHD group displayed scarce efficiencies of alerting and execution network concerning response time and response accuracy. Some compensatory mechanisms were noticeable in ADHD cases; the authors noted surpassing negativity in the occidental region on the right side than left, that is, the visual area of the right hemisphere. This study suggests that although there is an attention detriment with regard to the alerting network, there is also an anti-complement mechanism, reasonably present in the visual system that supplies visual signals that help in reforming clash between target and distraction and produce a fairly adequate response. One way or another, due to lower alertness efficiency, the ADHD patient’s brain might have re-circuit regional modality for perceptions and depiction in the visual cortex, which is unattached to the attention network. Here, it helps in ameliorating accuracy in response and ultimate performance in ADHD patients. Figure 3 shows the location of the visual cortex in the occipital region [39].

Limitations

There are some limitations of this study. The study only includes case-control studies in the English language from the last eight years; hence, we might have missed different types of studies in other languages and those published prior to 2012 related to this analysis. In the studies included, the sample size was relatively small, which could affect the results, limit their reliability, and deter the applicability of the results for the entire population with certainty.

## Conclusions

DMN and frontoparietal network are complementary in the execution of more natural tasks and salience network is selectively associated with reward processing, while others are unrelated. ADHD patients are more responsive to reinforcement related to monetary gain and loss and are more sensitive to larger rewards than smaller ones. ADHD-CT and typically developed controls were found to have similar responsiveness, while ADHD-I appeared to be insensitive to stimulus. The compensatory system might be located in the right visual cortex that reimburses for lower attention in patients. Future studies should focus more on these compensatory systems and their mechanism of action and how to make them more efficient. This study can help in implementing reinforcement learning in patients as an alternative to medication and the use of rewards for improving symptoms and increasing positive behavior in patients with ADHD.
